# Tissular Genomic Responses to Oral FB1 Exposure in Pigs

**DOI:** 10.3390/toxins14020083

**Published:** 2022-01-22

**Authors:** Léonie Dopavogui, Arnaud Polizzi, Anne Fougerat, Pascal Gourbeyre, Chloé Terciolo, Wendy Klement, Philippe Pinton, Joëlle Laffite, Anne-Marie Cossalter, Jean-Denis Bailly, Olivier Puel, Yannick Lippi, Claire Naylies, Hervé Guillou, Isabelle P. Oswald, Nicolas Loiseau

**Affiliations:** Toxalim (Research Centre in Food Toxicology), Université de Toulouse, INRAE, ENVT, INP-Purpan, UPS, 31300 Toulouse, France; leonie.dopavogui@inrae.fr (L.D.); arnaud.polizzi@inrae.fr (A.P.); Anne.Fougerat@inrae.fr (A.F.); pascal.gourbeyre@gmail.com (P.G.); chloe.terciolo@gmail.com (C.T.); klement.wendy@gmail.com (W.K.); philippe.pinton@inrae.fr (P.P.); joelle.laffitte@inrae.fr (J.L.); anne-marie.cossalter@inrae.fr (A.-M.C.); jean-denis.bailly@envt.fr (J.-D.B.); olivier.puel@inrae.fr (O.P.); yannick.lippi@inrae.fr (Y.L.); claire.naylies@inrae.fr (C.N.); herve.guillou@inrae.fr (H.G.)

**Keywords:** fumonisin, liver, jejunum, spleen, Peyer’s patches, swine

## Abstract

Fumonisin B1 (FB1) is a widespread mycotoxin produced by fungal *Fusarium* species—mainly in maize, one of the plants most commonly used for food and feed. Pigs and horses are the animal species most susceptible to this mycotoxin. FB1 exposure can cause highly diverse clinical symptoms, including hepatotoxicity, immunotoxicity, and intestinal barrier function disturbance. Inhibition of ceramide synthetase is a well-understood ubiquitous molecular mechanism of FB1 toxicity, but other more tissue-specific effects remain to be elucidated. To investigate the effects of FB1 in different exposed tissues, we cross-analyzed the transcriptomes of fours organs: liver, jejunum, jejunal Peyer’s patches, and spleen. During a four-week study period, pigs were fed a control diet or a FB1-contaminated diet (10 mg/kg feed). In response to oral FB1 exposure, we observed common biological processes in the four organs, including predominant and recurrent processes (extracellular matrix organization, integrin activation, granulocyte chemotaxis, neutrophil migration, and lipid and sterol homeostasis), as well as more tissue-specific processes that appeared to be related to lipid outcomes (cell cycle regulation in jejunum, and gluconeogenesis in liver).

## 1. Introduction

*Fusarium* spp. is among the most frequent fungal genus found on different cereal crops and causes dramatic economic losses and food safety concerns due to reduced cereal yield and quality [1]. Over the past decade, climate change has led to shifts in temperature and humidity weather conditions, which have favored *Fusarium* dissemination [2]. The variability in *Fusarium* spp. incidence generates a remodeling spectrum of mycotoxin exposure and a significant increase in cereal contamination, with potentially enhanced impacts on human and animal health. Contrasting weather conditions during cereal growing periods favor the proliferation of *F. verticillioides* and *F. proliferatum*. Global warming causes stronger climatic contrasts, which will likely lead to more frequent findings of high fumonisin concentrations in maize in temperate regions [3]. Fumonisins are the predominant mycotoxins produced by *Fusarium* spp., and fumonisin B1 is the most occurring and the most documented member of this family [4]. Since 2007, the European Union has set recommendations and regulations for the maximum level of the sum of fumonisins B1 (FB1) and B2 in animal (from 50 mg/kg feed for adult ruminants to 5 mg/kg feed for pigs) and human (from 4 mg/kg food for unprocessed maize to 0.2 mg/kg food for baby foods) foodstuffs [5,6].

FB1 exposure produces severe mycotoxicosis in pigs [4], with diverse clinical symptoms, most commonly including nephrotoxicity, hepatotoxicity [7], immunotoxicity [8,9], and intestinal barrier function disturbance [10]. To date, the molecular mechanism of FB1 toxicity has been predominantly studied through its inhibitory effect on ceramide synthase [11], which disrupts de novo sphingolipid metabolism. Indeed, FB1 and sphingoid long-chain bases share similar structural backbone features. Ceramide synthase inhibition increases free sphinganine levels, and decreases the content of complex sphingolipids and ceramides [10], resulting in an elevated ratio of free sphingoid bases (sphinganine/sphingosine; Sa/So) in several tissues (e.g., liver and intestine), in plasma, and in cell lines [12,13].

In the current study, we cross-analyzed changes in gene expression induced by FB1 exposure in four tissues: liver, jejunum, spleen, and jejunal Peyer’s patches. Our analysis was focused on characterizing the tissue distribution of the biological processes sensitive to FB1 exposure. During a four-week study period, pigs were fed a control diet or a FB1-contaminated diet around 10 mg/kg feed, a concentration of toxin known to induce physiological alteration in pigs [7,14,15] In response to oral FB1 exposure, common biological processes were observed in the four organs, involving predominant and recurrent processes (extracellular matrix organization, integrin activation, granulocyte chemotaxis, neutrophil migration, and lipid and sterol homeostasis), as well as more tissue-specific processes that appears to be related to lipid outcomes (cell cycle regulation in jejunum, and gluconeogenesis in liver).

## 2. Results

### 2.1. Genome-Wide Analysis of FB1 Effects on Gene Expression in Different Tissues

Genome-wide transcriptomic analysis was performed on the four tissues: liver, jejunum, Peyer’s patches, and spleen. Table 1 presents the number of significant genes (including non-annotated genes) showing an adjusted *p* value of <0.05 regarding the fold change (FC) threshold. Jejunum showed the largest number of modulated genes under FB1 exposure—followed by liver, Peyer’s patches, and spleen. When focusing on genes regulated with a FC greater than 1.5, FB1 exposure induced significant upregulation of 538 genes in jejunum, 241 in liver, 123 in Peyer’s patches, and 33 in spleen; and significant downregulation of 715 genes in jejunum, 194 in liver, 62 in Peyer’s patches, and 73 in spleen.

**Figure 1:**
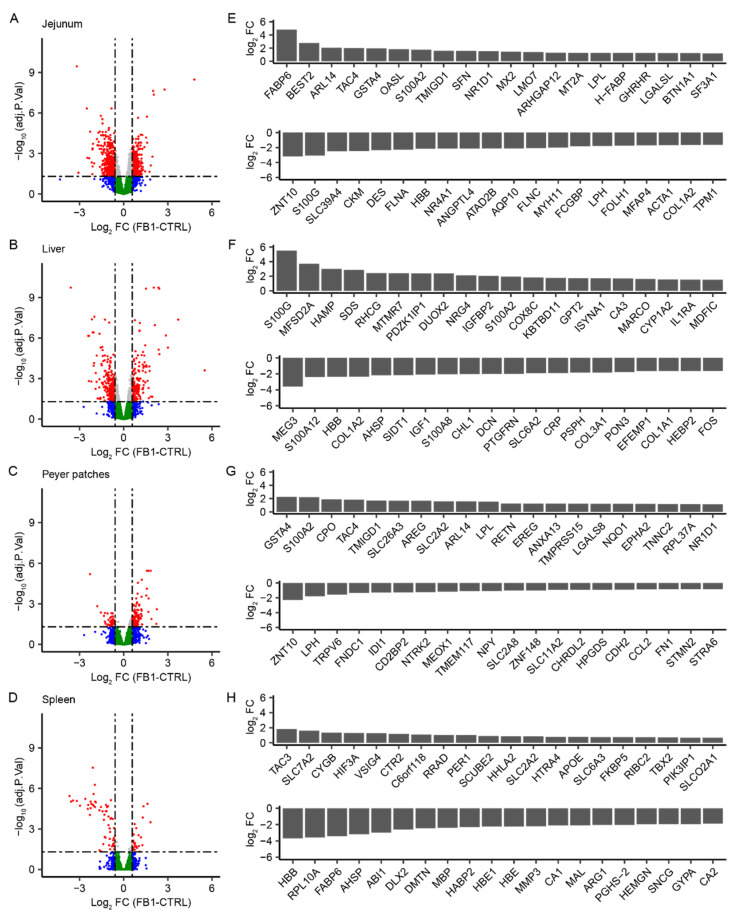
Comparison of transcriptomic gene expression profiling in the four studied tissues (jejunum, liver, Peyer’s patches, and spleen) under FB1 exposure. (**A**–**D**) Volcano plots show differences in gene expression between control (CTRL)- and FB1-exposed animals in: jejunum (**A**), liver (**B**), Peyer’s patches (**C**), and spleen (**D**). Red indicates *p* value < 0.05 and Log_2_ (fold change) >1.5. Grey indicates *p* value < 0.05 and Log_2_ (fold change) <1.5. Blue indicates *p* value > 0.05 and Log_2_ (fold change) > 1.5. Green indicates *p* value >0.05 and Log_2_ (fold change) <1.5. (**E**–**H**) Bar plots of the 20 upregulated and the 20 downregulated genes with the highest fold change in jejunum (**E**), liver (**F**), Peyer’s patches (**G**), and spleen (**H**).

According to this selection threshold, and considering the volcano plots of differences in gene expression under FB1-contaminated and control diets in each tissue type (Figure 1A–D), the transcriptomic effect of FB1 exposure included the most effects in jejunum and liver, with more downregulated genes and with more effects in jejunum than in liver. Moreover, under FB1 exposure, there were more upregulated genes in Peyer’s patches, and more downregulated genes in spleen. When comparing the significance and the level of gene induction observed in each tissue, the liver and jejunum responded to FB1 exposure with a higher fold change in gene expressions and stronger evidence compared to the spleen, which exhibited the most strongly downregulated genes, and the Peyer’s patches, which showed more moderate responses.

The top 20 upregulated and downregulated genes (with *p* values of <0.05) were plotted for each tissue (Figure 1E–H) in the order of decreasing log FC. Comparing the genes in these lists, 15 were regulated at least in two tissues. Seven genes were upregulated in both jejunum and Peyer’s patches: ARL14, TAC4, GSTA4, TMGD1, NR1D1, LPL, and S100A2. The HBB gene was downregulated in jejunum, liver, and spleen. FABP6, S100G, COL1A2, ZNT10, LPH, AHSP, and SLC2A2 were each regulated in two tissues. Determining the top-regulated genes enabled the identification of some important FB1-sensitive biological processes. The first identified process present in all tissues relates to the response to external chemicals or biotic stimulus, with the following genes: OASL, MX2, MT2A, FLNA, HBB, and TPM1 in jejunum; HAMP, S100A12, HBB, S100A8, CRP, and FOS in liver; SLC26A3, LPL, NR1D1, NTRK2, CCL2, and STMN2 in Peyer’s patches; and APOE and HBB in spleen. Another identified process was the regulation of cell proliferation and cell migration associated with an immune response, based on the following genes: S100A2, NR1D1, and NR4A1 in jejunum; DUOX2, IGFBP2, S100A12, IGF1, and S100A8 in liver; S100A2, EREG, LGALS8, and FN1 in Peyer’s patches; and VSIG4 and ARG1 in spleen. FB1 also appeared to impact extracellular structure organization and cellular adhesion, based on the following genes: ARHGAP12, DES, FLNA, HBB, FLNC, MYH11, MFAP4, ACTA1, COL1A2, and TPM1 in jejunum; COL1A2, IGF1, DCN, COL3A1, and COL1A1 in liver; and APOE and DMTN in spleen. Moreover, FB1 exposure apparently affected the global transport process in all tissues, as indicated by modulation of the following genes: MT2A and SLC39A4 in jejunum; MFSD2A, RHCG, CA3, HBB, and SLC6A2 in liver; SLC2A2 and SLC2A8 in Peyer’s patches; and SLC7A2, CYGB, SLC2A2, SLC6A3, SLCO2A1, HBB, CA1, and CA2 in spleen. Furthermore, FB1 also modulated lipid metabolic homeostasis in jejunum (FABP6, NR1D1, LPL, and ANGPTL4) and in spleen (APOE and FABP6). Finally, the oxidative stress metabolic process was affected through modulation of the following genes: DUOX2 and HBB in liver; GSTA4 and HPGDS in Peyer’s patches; and HBB, HBE, HBE1, and MMP3 in spleen.

**Figure 2:**
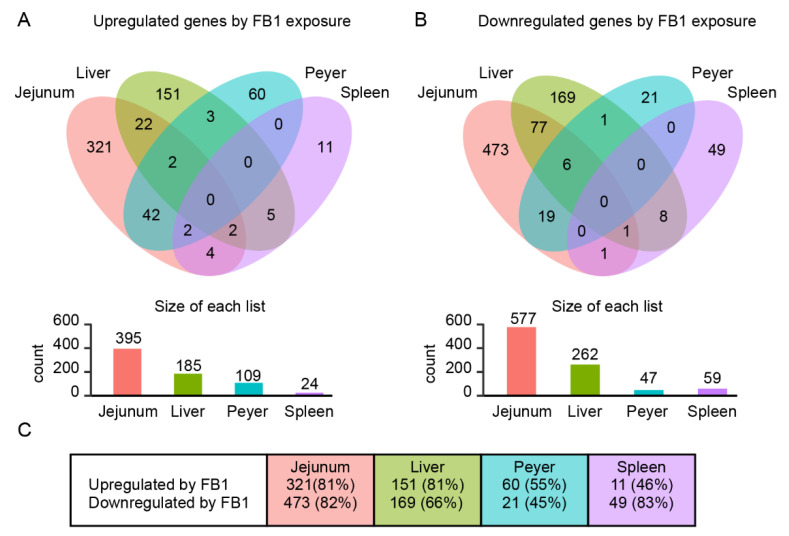
Overlapping of the significantly modulated gene sets between the four studied tissues, and characterization of the specificity ratio. Only annotated genes have been considered. (**A**,**B**) Venn diagram analysis of the upregulated (**A**) and the downregulated (**B**) genes (FC > 1.5 and *p* < 0.05) in the four tissues of FB1-exposed animals. (**C**) Recapitulative table of the numbers of genes specifically regulated in each tissue.

To investigate common or specific effects of FB1 exposure in the different tissues, Venn diagrams were drawn from only the annotated genes, for the upregulated and the downregulated genes (Figure 2). In the jejunum, 395 genes were upregulated and 577 downregulated in response to FB1 exposure, of which 321 upregulated genes (81.2%) and 473 downregulated genes (81.9%) were specifically regulated in jejunum. In the liver, 185 genes were upregulated and 262 downregulated in response to FB1 exposure, of which 151 upregulated genes (81.6%) and 169 downregulated genes (64.5%) were specifically regulated in liver. In Peyer’s patches, 109 were upregulated and 47 downregulated in response to FB1 exposure, of which 60 were upregulated (55.0%) and 21 downregulated (44.7%) specifically in Peyer’s patches. Finally, in the spleen, 24 genes were upregulated and 59 downregulated in response to FB1 exposure, of which 11 upregulated genes (45.8%) and 49 downregulated genes (83.0%) were specifically regulated in spleen. Considering these values, further investigation was conducted to confirm common, and to identify specific biological processes involved in responses to FB1 exposure in each tissue.

### 2.2. Identification of Common Fumonisin B1-Sensitive Biological Processes across Different Tissues

Based on Venn diagram analysis, gene ontology enrichment analysis was performed using Enrichr online tools, to identify common biological processes induced by FB1-exposure in the different tissues (Table 2). This analysis was conducted using the 82 common upregulated genes and 113 common downregulated genes. These results revealed upregulation of the following biological processes: triglyceride homeostasis, cholesterol homeostasis, sterol homeostasis, sulfur compound biosynthetic process, acylglycerol homeostasis, regulation of primary metabolic process, apoptotic mitochondrial changes, positive regulation of phospholipid biosynthetic process, negative regulation of defense response, and sodium ion transmembrane transport. However, the confidence in the enrichment analysis was low (adjusted *p* value > 0.01), except for triglyceride homeostasis, cholesterol homeostasis, and sterol homeostasis. On the other hand, analysis of downregulated genes revealed the involvement of the following biological processes with high confidence (adjusted *p* value < 0.01): extracellular matrix organization, collagen fibril organization, supramolecular fiber organization, cellular protein metabolic process, integrin activation, positive regulation of cellular process, granulocyte chemotaxis, positive regulation of macromolecule metabolic process, endothelial cell migration, and neutrophil migration.

**Figure 3:**
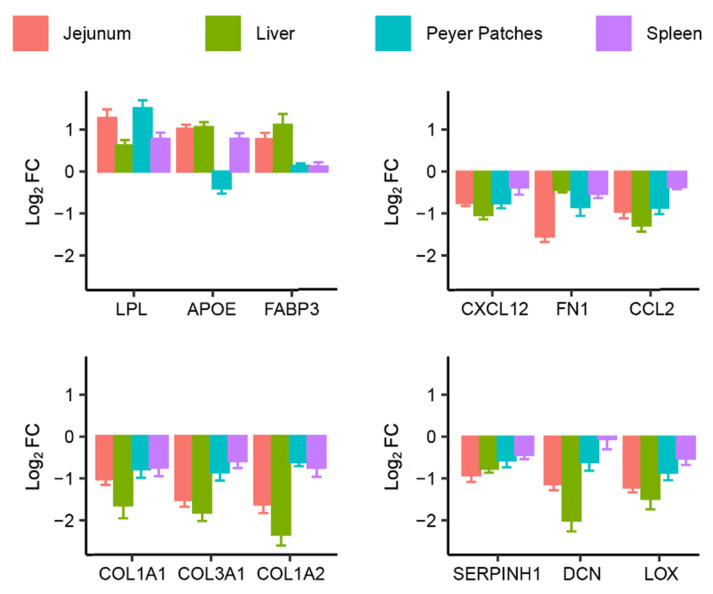
Effect of FB1 exposure on pertinent genes expression in the four studied tissues (jejunum, liver, Peyer’s patches, and spleen). Bar plots of some typical upregulated and downregulated genes representative of common biological processes present in the four studied tissues. Colors indicate the results according the tissue. Plotted data represent the mean ± SD of six animals.

Based on this enrichment analysis, and the most significant changes in mRNA expression levels, a set of 3 upregulated genes (LPL, APOE, and FABP3) and 9 downregulated genes (COL1A1, COL3A1, COL1A2, SERPINH1, DCN, LOX, CXCL12, FN1, and CCL2) were selected to be representative of the different biological processes involved. Their relative mRNA gene expressions were plotted according to tissue (Figure 3). Among the upregulated genes representing lipid homeostasis, LPL was the most pertinent gene in jejunum and Peyer’s patches, whereas FABP3 was more pertinent in liver. Among the six downregulated genes representing extracellular matrix organization, the three most pertinent and sensitive genes were COL1A1, COL3A1, and COL1A2. Additionally, CXCL12, FN1, and CCL2 were important in terms of their sensitivity and their implication in the respective biological processes: integrin activation and granulocyte chemotaxis.

### 2.3. Identification of Tissue-Specific Biological Processes Sensitive to FB1 Exposure

**Figure 4:**
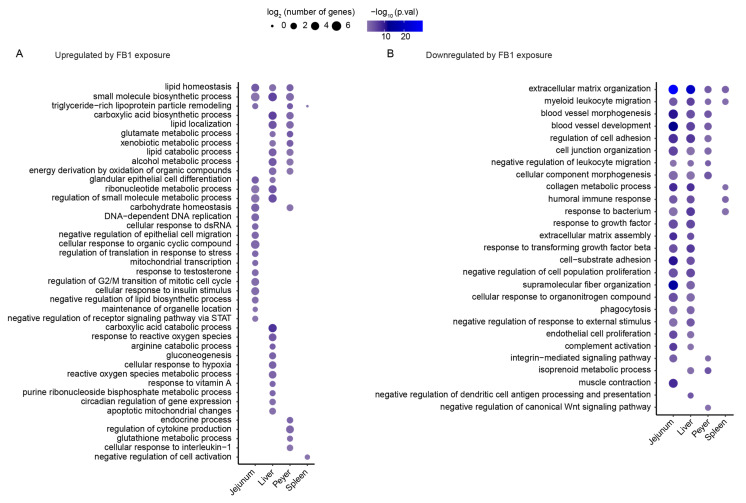
Determination of specific biological processes found in the four studied tissues (jejunum, liver, Peyer’s patches, and spleen). Balloon plots present the results of the hypergeometric test performed on biological processes identified from gene ontology analysis based on upregulated genes (**A**), and downregulated genes (**B**). Sizes indicate the Log_2_ (number of genes) associated with the indicated biological process. Colors indicate the hypergeometric test -Log_10_ (*p* value), from violet for low-confidence results to shiny blue for high-confidence results.

To explore the tissue-specific responses to FB1 exposure, Gene ontology analysis was performed on the whole set of significant differentially expressed genes, using metascape online tools. Their hypergeometric *p* values were determined in each tissue and expressed through a balloon plot. As shown in Figure 4, most biological processes identified from downregulated genes were shared between 2–4 tissues. Extracellular matrix organization and myeloid leukocyte migration were identified in all tissues. Only three biological processes were entirely tissue-specific—muscle contraction, negative regulation of dendritic cell antigen processing and presentation, and negative regulation of the canonical Wnt signaling pathway—with relatively good-to-low confidence.

In contrast, only one-third (14 of 41) of biological processes identified from upregulated genes were shared between several tissues, including lipid homeostasis and metabolic processes. Most identified biological processes were tissue-specific: 12 specific to jejunum, 10 to liver, 4 to Peyer’s patches, and 1 to the spleen.

## 3. Discussion

Dietary exposure to FB1 causes various clinical signs, most frequently including nephrotoxicity [24], hepatotoxicity [25], immunotoxicity [8,9], disturbances of intestinal barrier function [26], and microbiota dysbiosis [27]. Pigs are among the mammalian species most susceptible to FB1 toxicity [28]. In the present study, we focused on transcriptomic changes in several porcine tissues: jejunum, liver, Peyer’s patches, and spleen. Jejunum and liver are two major targets of FB1, due to their level of exposure and their respective functions of providing a barrier against food contaminants and as a detoxification organ. Peyer’s patches and spleen are also particularly affected, being lymphoid organs involved in the FB1-induced immune response.

The experimental design for FB1 exposure of animals was previously reported in 2017 [16]. That prior experiment revealed that the physiological effects of FB1 induce significant changes in the weight gain of animals receiving the FB1-contaminated diet compared to control, associated with decreased feed consumption. This phenomenon is common among pigs exposed to contaminated feed and has been reported in animals exposed to 8 mg FB1/kg feed [29]. To confirm FB1 exposure, the Sphingoid base ratio (Sa/So) has been measured in liver and has been shown to be significantly higher in animals receiving a FB1-contaminated diet. This ratio is considered a pertinent marker of FB1 exposure and is increased in FB1-exposed animals [30,31,32]. Furthermore, a decreased jejunum villi length has been reported in animals fed FB1 and reflects an inhibitory effect of FB1 on cell differentiation [33]. All observed physiological parameters in our study were in good agreement with standard FB1-contaminated feed exposure.

As shown in the summary of significant genes (Table 1) and the volcano plots (Figure 1), the number and the fold-change ratio of genes that were significantly modulated were greater in jejunum followed by liver, compared to in Peyer’s patches or spleen. This difference may be due to the tissue concentration in FB1. Indeed, after oral exposure, jejunum is one of the most exposed tissues. Under sub-acute or sub-chronic exposure to low-dose FB1 (3.7–4.1 mg/kg feed), the toxic effect is first observed in the jejunal proximal part of the intestine, and characterized by histopathological features, such as enterocyte flattening, apical necrosis of villi, and increased lesional score [7]. Upon subacute exposure to the toxin at 12.2 mg/kg feed for 28 days, in addition to morphological changes in jejunum, histological lesions appear in both the liver and spleen—including nuclear megalocytosis and cytoplasmic vacuolization of hepatocytes, and the presence of lymphocytic apoptosis bodies and follicular depletion in the spleen [6]. Acute exposure to 20–30 mg/kg feed for 9–10 days is necessary to observe a reduction in the number of antigen-specific IgM antibody-secreting cells in Peyer’s patches and in jejunum [8]. This acute high level of exposure also induces weight loss of the spleen and weight increase of the liver [18,34].

Moreover, analyzing the top 20 regulated genes in each tissue (Figure 1E–H) revealed that the most important FB1-sensitive biological processes present in all tissues were related to responses to external chemicals or biotic stimulus, the regulation of cell proliferation, and the regulation of cell migration associated with the immune response. These results are in accordance with previously published results demonstrating that under subchronic exposure to FB1, inflammatory responses show decreased IL1-beta and IL6 in the spleen [12] and jejunum [21]. Moreover, acute FB1 exposure is reportedly associated with a lower number of antigen-specific IgM antibody-secreting cells in Peyer’s patches and jejunum [8]. This is also in accordance with previous results from our group showing that FB1 decreases the gene expressions of TLR4 and MYD88 [16] and disturbs development of the humoral immune response and antibody production [22,23].

Other identified FB1-sensitive biological processes involve extracellular structure organization and cellular adhesion. These results are in agreement with previously published findings that FB1 exposure affects matrix cell organization through the integrin and actin pathways [16]. Accordingly, it has also been reported that chronic FB1 exposure is associated with morphological changes in the jejunum, including atrophy and fusion of villi, and with reduced expression of adherent junction proteins (E-cadherin and occludin), suggesting higher permeability of the epithelium [21,33,35]. Our results also confirmed that FB1 affects the global transport process, as previously reported in pigs under acute FB1 exposure [20]. This study showed that FB1 exposure led to higher ion secretion, sodium-dependent glucose absorption, and theophylline-induced secretion of the jejunal mucosa, suggesting altered jejunal permeability, and thus intestinal barrier integrity. Finally, our analysis of the top 20 genes modulated by FB1 also confirmed the involvement of lipid metabolic homeostasis [16,17], and oxidative stress metabolic process, as was already established based on the increased production of stress proteins in the gastrointestinal tract [18].

The Venn diagram of the genes significantly modulated by FB1 exposure in each tissue (Figure 2) reveals that over 50–80% of the modulated genes were specific to the considered tissue. Moreover, gene ontology enrichment analysis revealed that whereas most of the significantly downregulated genes were involved in common biological processes, the upregulated genes were more often associated with tissue-specific biological processes.

To focus on common biological processes, the first gene ontology enrichment analysis was processed using the set of genes significantly regulated by FB1 exposure in more than one tissue. The top ten biological processes induced by FB1 exposure, based on upregulated genes and downregulated genes, are presented in Table 2. We also identified previous published data describing associations between the upregulated genes and lipid homeostasis and other lipid-related biological processes [16]. It has also been previously shown that some of the downregulated genes are associated with cellular organization and the immune response. Using this enrichment, we characterized two upregulated genes (LPL and FABP3) and six downregulated genes (CXCL12, FN1, CCL2, COL1A1, COL3A1, and COL1A2) as being involved in biological processes pertinent to the toxicological effects observed in the tissues under FB1 exposure (Figure 3).

To evaluate the representativeness of the common identified biological processes, and to expand our analysis for the characterization of tissue-specific biological processes, the gene ontology enrichment analysis was next performed on the whole set of genes significantly modulated by FB1 exposure. The hypergeometric *p* value was determined for each tissue to enable presentation of the relevant results as a balloon plot (Figure 4). The biological processes identified from downregulated genes, and shared between the four tissues, included extracellular matrix organization and myeloid leukocyte migration. Moreover, other biological processes present in two or three tissues are related to the immune response or to cell adhesion or organization. Similarly, biological process linked to lipid homeostasis were identified from upregulated genes, and shared between the jejunum, liver, and Peyer’s patches.

Whereas common biological processes were prevalently enriched from downregulated genes analysis, three-quarters of the biological processes identified from gene ontology enrichment of upregulated genes were tissue-specific. Indeed, among the 27 specific biological processes identified from upregulated genes, most were specific to the jejunum (12) or the liver (10). Looking closer at these results, the 12 biological processes specifically enriched from jejunum have been closely associated with the regulation of cell proliferation. These findings suggest that FB1 exposure has a specific effect in epithelial cells, relating to the cellular proliferation involved in carcinogenesis or in response to DNA damage, as already suggested by several studies examining the inhibitory effect of S1P on the HDAC/PI3K/Akt pathway [19]. Analysis of the 10 biological processes specifically enriched in the liver under FB1 exposure suggests that the modulation is related to the glutamine and alanine metabolism associated with gluconeogenesis homeostasis.

Altogether, these results extend and further confirm that FB1 exposure has major impacts on the expressions of genes related to cellular organization, immune response, and lipid metabolic processes across four different organs. Our data additionally suggest that FB1 exposure induces ubiquitous biological processes through multiple tissue-specific gene expression responses.

## 4. Conclusions

Our present results indicate that in the jejunum, liver, and Peyer’s patches, the effects of FB1 exposure are associated with biological processes related to extracellular matrix organization and immune response process, as shown by downregulation of the COL1A1, COL3A1, COL1A2, CXCL12, FN1, and CCL2 genes. Our findings also confirmed that FB1 exposure was related to modulation of lipid homeostasis, as revealed by the upregulation of LPL and FABP3. Moreover, FB1 had sphingolipid-related tissue-specific effects, such as the modulation of genes associated with the cell cycle in jejunum, and the modulation of gluconeogenesis-related genes in the liver. Further work is needed to elucidate the molecular mechanisms underlying these changes in gene expression, e.g., whether they are cell-autonomous and directly related to the effect of FB1 exposure on ceramide synthesis. Our work suggests that feed contaminated with FB1 can affect pig’s health and possibly growth, through modulation of immunity and metabolism

## 5. Materials and Methods

### 5.1. Animals

Twelve castrated male pigs (Pietrain/Duroc/Large-white) weaned at 28 days of age were obtained from a local farm (Gaec de Calvignac, St Vincent d’Autejac, France). They were acclimatized in the animal facility of the INRAE ToxAlim Unit (Toulouse, France) for one week prior to being used in experimental protocols. Animals were given ad libitum access to water and feed. The experiments were carried out in accordance with European Guidelines for the Care and Use of Animals for Research Purposes (accreditation number APAFIS#5917-2016070116429578 v3).

### 5.2. Feeding Trial and Sampling

Diets were manufactured at INRAE facilities in Rennes (France) and formulated according to the energy and amino acid requirements for piglets. Two different batches were prepared, one control batch and one batch artificially contaminated with mycotoxin, as previously described [36]. For the 4-week study period, 6 pigs were fed ad libitum the control diet, and 6 pigs were fed ad libitum the contaminated diet (10.2 mg FB1 + 2.5 mg FB2 + 1.5 mg FB3/kg). The feed was analyzed for mycotoxin content (Labocea, Ploufragan, France). Deoxynivalenol and zearalenone were naturally present in the raw cereals used to prepare both batches control and artificially contaminated diets, at concentrations of 0.12 and 0.015 mg/kg feed, respectively. All other mycotoxins were below the limits of detection, including aflatoxins, T-2 toxin, HT-2 toxin, and ochratoxin A.

Animals were slaughtered by electro-narcosis prior to exsanguination. Following euthanasia, samples were collected from jejunum, liver, Peyer’s patches, and spleen. These samples were snap-frozen in liquid nitrogen and stored at −80 °C for transcriptome analysis.

### 5.3. Gene Expression Analysis by Microarray

Total RNA was extracted in lysing matrix D tubes (MP Biomedicals, Illkirch, France) containing guanidine thiocyanate-acid phenol (Eurobio). Transcriptome profiles were obtained at the GeT-TRiX facility (GénoToul, Génopole Toulouse Midi-Pyrénées). RNA quality was confirmed using the Agilent RNA 6000 Nano Kit, with the Agilent Bioanalyzer 2100. Reverse transcription was performed as previously described [16]. This experiment was conducted using microarray GPL16524 (Agilent Technology, Santa Clara, CA, USA, 8 × 60 K), which comprised 43,603 spots derived from the 44K (V2:026440 design) Agilent porcine-specific microarray and completed with 9532 genes from adipose tissue, 3776 genes from the immune system, and 3768 genes from skeletal muscle [37]. For each sample, Cyanine-3 (Cy3)-labeled cRNA was prepared as previously described [38], from 200 ng total RNA using the One-Color Quick Amp Labelling kit (Agilent technologies) according to the manufacturer’s instructions. Purification was performed using Agencourt RNAClean XP (Agencourt Bioscience Corporation, Beverly, MA, USA). Dye incorporation and cRNA yield were checked using the DropsenseTM 96 UV/VIS droplet reader (Trinean, Gent, Belgium). Cy3-labeled cRNA (600 ng) was hybridized on the microarray slides following the manufacturer’s instructions. Immediately after washing, the slides were scanned on an Agilent G2505C Microarray Scanner with Agilent Scan Control A.8.5.1 software. The fluorescence signal was extracted using Agilent Scan control v10.10.1.1 with default parameters. All subsequent data analyses were performed using R (www.rproject.org (last accessed on 21 January 2022)), with packages from Bioconductor (www.bioconductor.org (last accessed on 21 January 2022)). Raw data (median signal intensity) were filtered, log_2_ transformed, and normalized using Smooth quantile normalization. Microarray data and experimental details are available in NCBI’s Gene Expression Omnibus [39] and are accessible with the GEO Series accession number GSE189198 (https://www.ncbi.nlm.nih.gov/geo/query/acc.cgi?token=mfcbemystrilpml&acc=GSE189198 (last accessed on 21 January 2022)).

### 5.4. Bioinformatic Data Analysis

Venn analysis was performed to identify common or specific genes with modulated expression levels in different organs. Data were processed using R (www.r-project.org (last accessed on 21 January 2022)). Gene ontology enrichment of biological processes was performed using either Enrichr online tools [40,41] or Metascape online tools [42] (accounting for the microarray background list of genes for a better analysis) to determine common and specific regulated biological processes, respectively.

### 5.5. Statistical Analysis

Gene ontology enrichment results were verified by performing a hypergeometric test to determine the confidence and the specificity of the results for each tissue through the *p* value determination. Subsequently, the results were plotted as a balloon plot.

## Figures and Tables

**Table 1:** Summary of significant genes (including non-annotated genes) regarding the fold change (FC) threshold for an adjusted *p* value < 0.05. To determine which biological processes are specifically involved in each FB1-exposed tissue, the subsequent analyses focused on genes with a FC > 1.5.

Comparison	*P* < 0.05	FC > 1.0	FC > 1.2	FC > 1.5	FC > 2	FC > 4	FC > 6	FC > 10
Jejunum FB1- Jejunum Ctrl	Up	1536	1521	538	71	7	4	1
	Down	1172	1168	715	193	17	3	0
	Total	2708	2689	1253	264	24	7	1
Liver FB1- Liver Ctrl	Up	477	477	241	81	10	4	2
	Down	454	454	194	102	12	2	2
	Total	931	931	435	183	22	6	4
Peyer FB1- Peyer Ctrl	Up	154	154	123	38	3	0	0
	Down	73	73	62	17	2	0	0
	Total	227	227	185	55	5	0	0
Spleen FB1- Spleen Ctrl	Up	53	53	33	10	1	0	0
	Down	81	81	73	44	19	7	3
	Total	134	134	106	54	20	7	3

**Table 2:** The top 10 biological processes in terms of Gene Ontology enrichments performed using Enrichr online tools, from common upregulated genes (**a**) and the common downregulated genes (**b**).

Term	Overlap	Adjusted *p* Value	Genes	Ref
Common upregulated genes (**a**)				
triglyceride homeostasis (GO:0070328)	4/31	0.003	HNF4A; NR1H4; LPL; APOE	[16,17]
cholesterol homeostasis (GO:0042632)	5/71	0.003	FABP3; HNF4A; LPL; APOE; NR1D1	[16,17]
sterol homeostasis (GO:0055092)	5/72	0.003	FABP3; HNF4A; LPL; APOE; NR1D1	[16,17]
sulfur compound biosynthetic process (GO:0044272)	5/113	0.019	GSTA4; CTH; MGST1; GCLM; PAPSS2	[18]
acylglycerol homeostasis (GO:0055090)	3/25	0.023	HNF4A; LPL; APOE	[16,17]
regulation of primary metabolic process (GO:0080090)	5/130	0.027	NQO1; HNF4A; FASN; APOE; NR1D1	[16,17,18]
apoptotic mitochondrial changes (GO:0008637)	3/33	0.036	BAD; ATP2A1; SFN	[19]
positive regulation of phospholipid biosynthetic process (GO:0071073)	2/7	0.036	FABP3; NR1H4	[16,17]
negative regulation of defense response (GO:0031348)	5/85	0.036	NR1H4; APOE; NR1D1; KLF4	[16,19]
sodium ion transmembrane transport (GO:0035725)	5/87	0.036	SCN8A; SCNN1A; ATP1B1; SLC6A3	[20]
Common downregulated genes (**b**)				
extracellular matrix organization (GO:0030198)	27/300	5.46 × 10^−22^	COL15A1; COL16A1; SPARC; ECM2; COL14A1; COL12A1; HTRA1; DPT; LAMC1; LOXL2; FLRT2; SERPINH1; ITGAV; POSTN; FN1; DCN; COL1A1; GREM1; COL3A1; BMP1; COL1A2; LOX; MMP16; COL4A1; COL5A2; COL6A3; FBN1	[16]
collagen fibril organization (GO:0030199)	16/89	2.63 × 10^−17^	COL15A1; COL16A1; COL14A1; COL12A1; DPT; LOXL2; GREM1; COL1A1; COL3A1; BMP1; COL1A2; LOX; COL4A1; COL5A2; SERPINH1; COL6A3	[16]
supramolecular fiber organization (GO:0097435)	22/351	9.89 × 10^−15^	COL15A1; COL16A1; TNXB; COL14A1; COL12A1; TPM1; STMN2; DPT; LOXL2; COL1A1; GREM1; EFEMP2; COL3A1; BMP1; COL1A2; ACTC1; LOX; COL4A1; COL5A2; SERPINH1; COL6A3; EMILIN1	[16]
cellular protein metabolic process (GO:0044267)	12/147	7.14 × 10^−4^	IL6; RCN1;L GALS1; LOX; SDC2; IGFBP2; FN1; IGFBP7; LAMC1; FSTL1; LOXL2; FBN1	[12,16,21]
integrin activation (GO:0033622)	5/8	0.0012	COL16A1; CXCL12; FN1	[16]
positive regulation of cellular process (GO:0048522)	13/625	0.0047	SLC30A2; TPM1; FN1; HBB; GREM1; IL6; CXCL12; CXCR2; ITGAV; S100A9; SOX4; S100A8; TLR2	[12,16,21]
granulocyte chemotaxis (GO:0071621)	5/73	0.0047	CXCR2; CCL2; S100A12; S100A9; S100A8	[19,22,23]
positive regulation of macromolecule metabolic process (GO:0010604)	10/384	0.0047	GREM1; ACTA2; GJA1; IL6; ACTC1; FN1; EMILIN1; ANK3; ROBO1; TLR2	[12,16,21]
endothelial cell migration (GO:0043542)	4/39	0.0047	PLXND1; FAP; FSTL1; LOXL2	[19]
neutrophil migration (GO:1990266)	5/77	0.0049	CXCR2; CCL2; S100A12; S100A9; S100A8	[19,22,23]

## Data Availability

The data presented in this study are openly available in this article.
